# Supplementary material for: Early enhanced control of Plasmodium yoelii infection in IL-10-deficient mice is independent of IFN-γ, IL-12, and the humoral response
Source: Infect Immun. 2026 Aug 6;94(9):e00196-26. doi: 10.1128/iai.00196-26 (PMC13552106; doi:10.1128/iai.00196-26)
Supplement: Supplementary file 1 [file iai.00196-26-s0001.pdf]

Figure S1

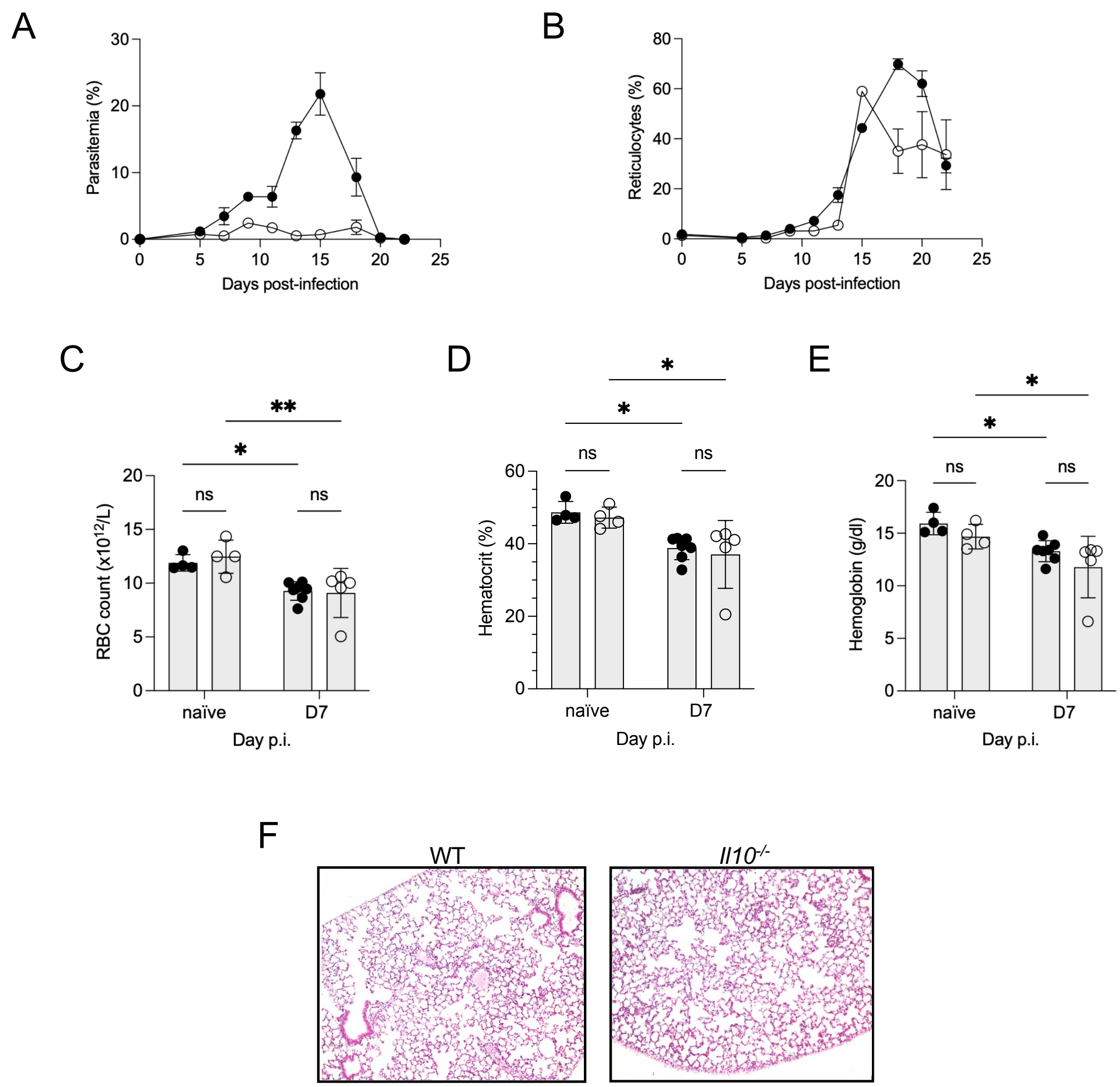

Fig S1. WT and *Il10*$^{-/-}$ mice were infected i.p. with 10^5 *P. yoelii* 17X pRBCs. (**A**) Representative parasitemia curve of the primary infection. (**B**) Frequency of reticulocytes out of total RBCs during the primary infection. Uninfected reticulocyte counts were measured via flow cytometry. (**A,B**) Data is representative of three independent experiments with 4-5 mice per group. (**C**) RBC count, (**D**) hemoglobin, and (**E**) hematocrit values were determined by analyzing blood collected from naïve and Day 7 infected mice using a Vetscan Hematology Analyzer. (**F**) H&E-stained lung sections from D14 p.i. Microscopic images were taken at 10× magnification. (**C-E**) A two-way ANOVA determined significance. *$p < 0.05$, ** $p < 0.01$, ns = non-significant.

Figure S2

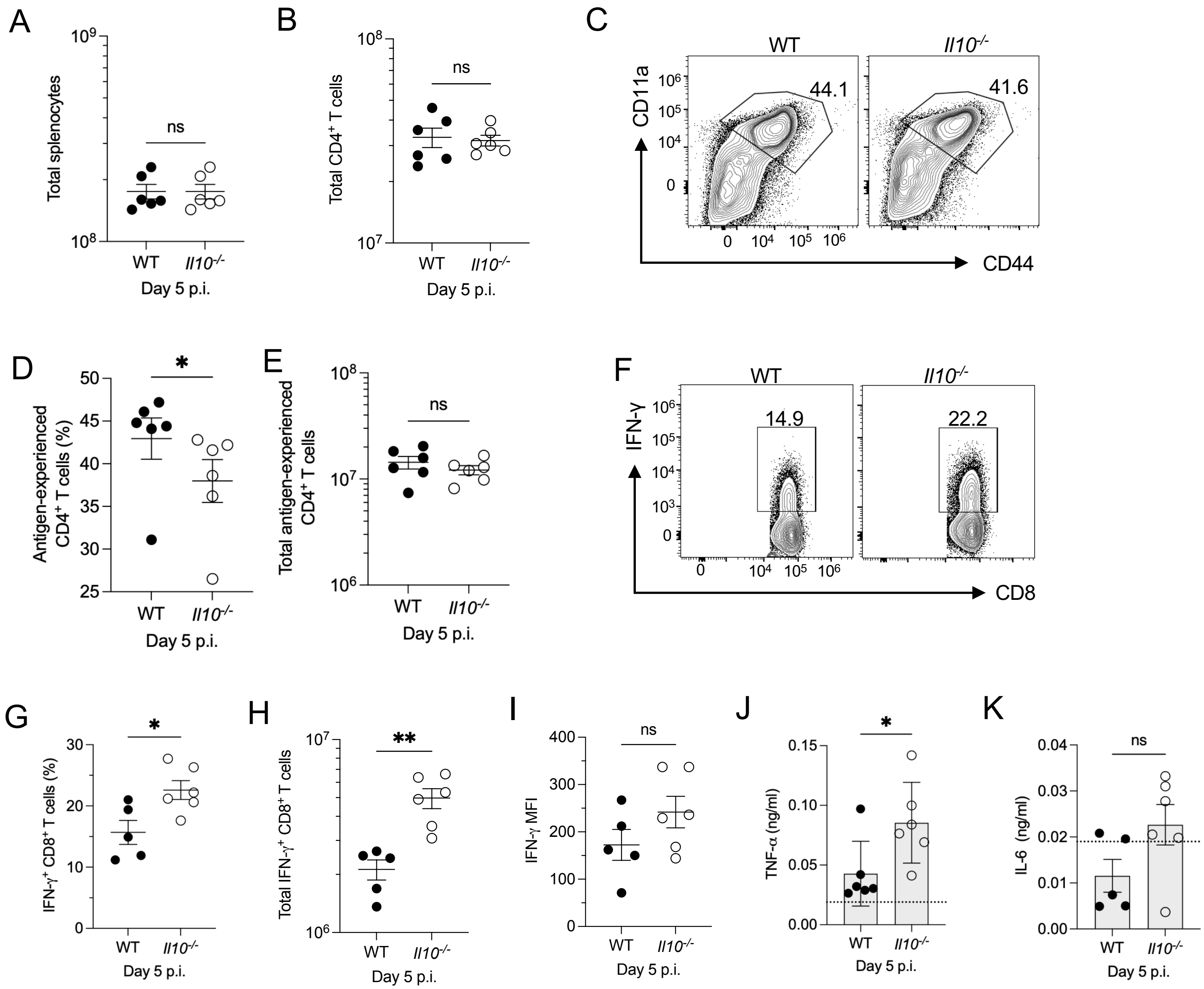

Fig S2. WT and *Il10*$^{-/-}$ mice were infected i.p. with 10^5 *P. yoelii* 17X pRBCs. (**A**) Total splenocytes at day 5 p.i. (**B**) Total CD4$^+$ T cells in the spleen at day 5 p.i. **(C)** Representative flow plots of live, single cell, antigen-experienced CD11a^{hi}CD44^{hi} CD4$^+$ T cells in the spleen at day 5 p.i. (**D**) Frequency out of the total CD4$^+$ T cell population and (**E**) total number of antigen-experienced CD4$^+$ T cells. (**F**). Representative flow plots of live, single-cell, IFN-γ$^+$ CD8$^+$ T cells at day 5 p.i. in the spleen. (**G**) Frequency of IFN-γ$^+$ CD8$^+$ T cells out of total CD8$^+$ T cells. (**H**) Total IFN-γ$^+$ CD8$^+$ T cells in the spleen. (**I**) Median fluorescent intensity (MFI) of IFN-γ in CD8$^+$ T cells. (**J**) Serum IL-6 and (**K**) TNF-α at day 5 p.i. determined by Biolegend™ multiplex assay. Data is representative of two independent experiments, with 4-6 mice per group. (**A**, **B, D, E, G, H, I, J, K**) A non-parametric Mann-Whitney t-test determined significance. *$p < 0.05$, ** $p < 0.01$, ns = non-significant.

Figure S3

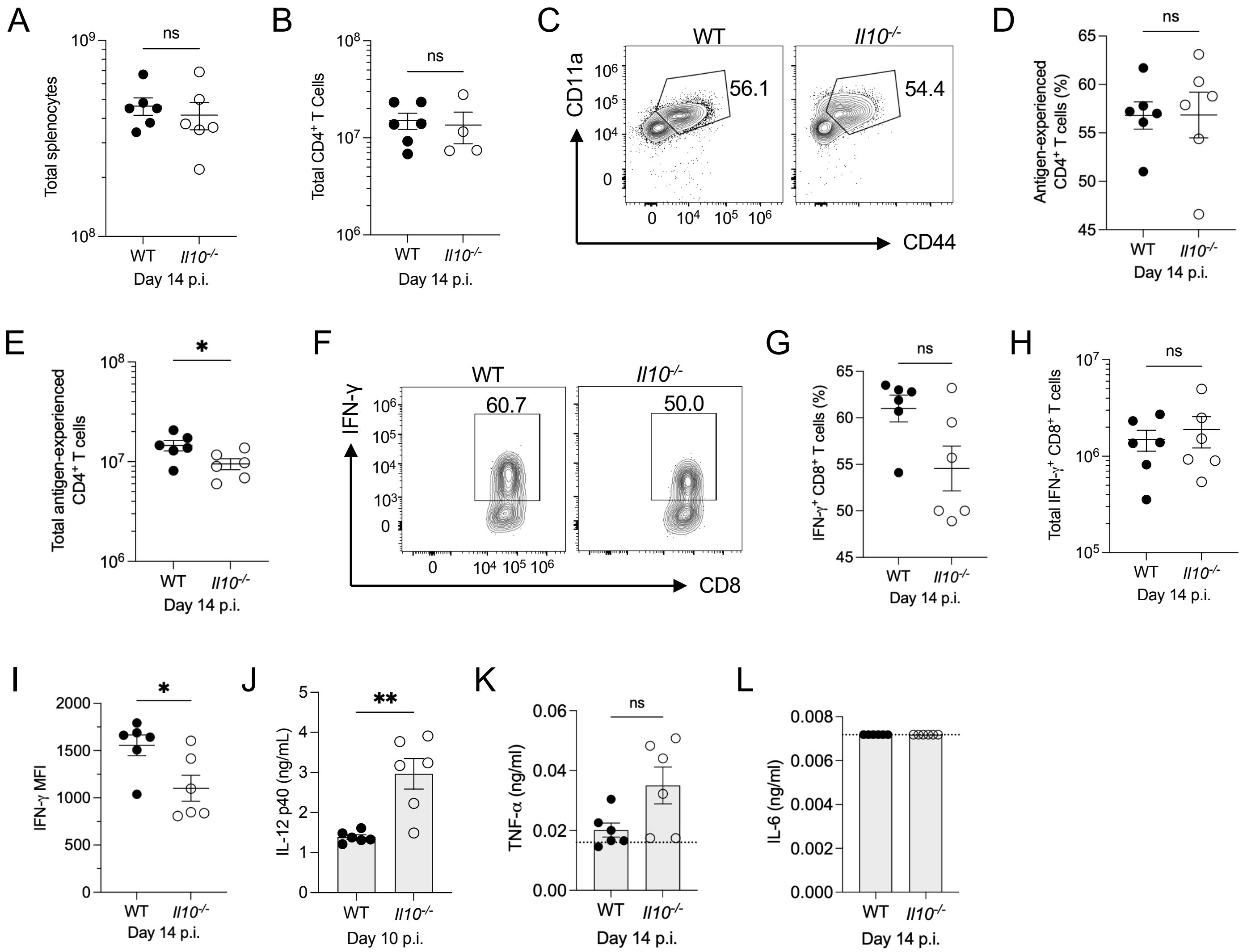

Fig S3. WT and *Il10*$^{-/-}$ mice were infected i.p. with 10^5 *P. yoelii* 17X pRBCs. (**A**) Total splenocytes at day 14 p.i. (**B**) Total CD4$^+$ T cells in the spleen at day 14 p.i. **(C)** Representative flow plots of live, single-cell, antigen-experienced CD11a^{hi}CD44^{hi} CD4$^+$ T cells in the spleen at day 14 p.i. (**D**) Frequency of antigen-experienced CD4$^+$ T cells out of the total CD4$^+$ T cell population. (**E**) Total activated CD4$^+$ T cells in the spleen. (**F**). Representative flow plots of live, single-cell, IFN-γ^+ CD8$^+$ T cells at day 14 p.i. in the spleen. (**G**) Frequency of IFN-γ^+ CD8$^+$ T cells out of total CD8$^+$ T cells. (**H**) Total IFN-γ^+ CD8$^+$ T cells in the spleen. (**I**) Median fluorescent intensity (MFI) of IFN-γ in CD8$^+$ T cells. (**J**) Serum IL-12p40 at day 10 p.i. determined by ELISA. (K) Serum IL-6 and (**L**) TNF-α at day 14 p.i. determined by Biolegend™ multiplex assay. Data is representative of two independent experiments, with 4-6 mice per group. (**A**, **B, D, E, G, H, I, J, K, L**) A non-parametric Mann-Whitney t-test determined significance. **p* < 0.05, ns = non-significant. Dotted lines represent the limit of detection.

Figure S4

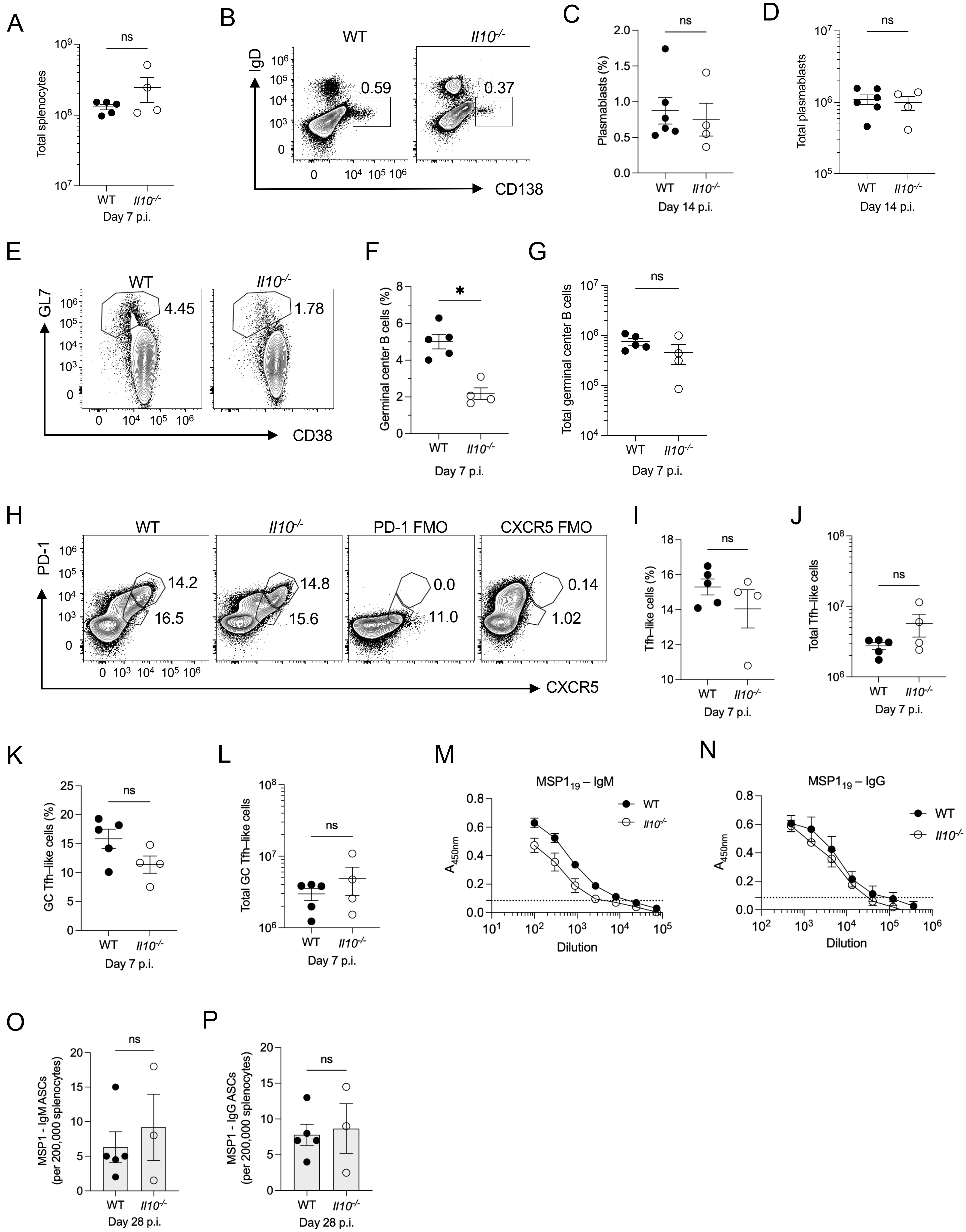
A
Total splenocytes
ns
WT
Il10-/-
Day 7 p.i.
B
WT
Il10-/-
IgD
CD138
0.59
0.37
C
Plasmablasts (%)
ns
WT
Il10-/-
Day 14 p.i.
D
Total plasmablasts
ns
WT
Il10-/-
Day 14 p.i.
E
WT
Il10-/-
GL7
CD38
4.45
1.78
F
Germinal center B cells (%)
*
WT
Il10-/-
Day 7 p.i.
G
Total germinal center B cells
ns
WT
Il10-/-
Day 7 p.i.
H
WT
Il10-/-
PD-1 FMO
CXCR5 FMO
PD-1
CXCR5
14.2
16.5
14.8
15.6
0.0
11.0
0.14
1.02
I
Tfh-like cells (%)
ns
WT
Il10-/-
Day 7 p.i.
J
Total Tfh-like cells
ns
WT
Il10-/-
Day 7 p.i.
K
GC Tfh-like cells (%)
ns
WT
Il10-/-
Day 7 p.i.
L
Total GC Tfh-like cells
ns
WT
Il10-/-
Day 7 p.i.
M
MSP1 19 – IgM
WT
Il10-/-
A450nm
Dilution
N
MSP1 19 – IgG
WT
Il10-/-
A450nm
Dilution
O
MSP1 - IgM ASCs (per 200,000 splenocytes)
ns
WT
Il10-/-
Day 28 p.i.
P
MSP1 - IgG ASCs (per 200,000 splenocytes)
ns
WT
Il10-/-
Day 28 p.i.

Fig S4. WT and *Il10*$^{-/-}$ mice were infected i.p. with 10^5 *P. yoelii* 17X pRBCs. (**A**) Total splenocytes on D7 and (**B**) Representative flow plots of live, single-cell, CD138$^+$IgD$^-$ splenic plasmablasts 14 days p.i. (**C**) Frequency of plasmablasts out of CD3$^-$, Ter119$^-$, CD11c$^-$, CD11b$^-$ cells. (**D**) Total plasmablasts in the spleen. (**E**) Representative flow plots of live, single-cell splenic CD38$^-$GL7$^+$ GC B cells at 7 days p.i. (**F**) Frequency of splenic GC B cells out of total CD19$^+$B220$^+$ B cells. (**G**) Total GC B cells in the spleen. (**H**) Representative flow plots of live, single-cell, PD-1$^+$CXCR5$^+$ Tfh-like cells in the spleen at day 7 p.i. FMO controls were used to set gates. (**I**) Frequency of Tfh-like cells out of CD4$^+$ T cells. (**J**) Total Tfh-like cells in the spleen. (**K**) Frequency of GC Tfh cells out of CD4$^+$ T cells. (**L**) Total GC Tfh cells in the spleen at day 7 p.i. (**M**) Serum IgM and (**N**) IgG titers against MSP-1_{19} at day 28 p.i. (**O**) Total MSP1_{19}–IgM and (**P**) MSP1_{19}–IgG secreting cells in the spleen per 200,000 splenocytes at day 28 p.i. detected by ELISpot. (**A-L**) Data is representative of three independent experiments, with 4-5 mice per group. (**A, C, D, F, G, I, J, K, L, N, P**) A non-parametric Mann-Whitney t-test determined significance. *$p < 0.05$, ns = non-significant. The dotted line in **M** and **N** represents the limit of detection.

A B

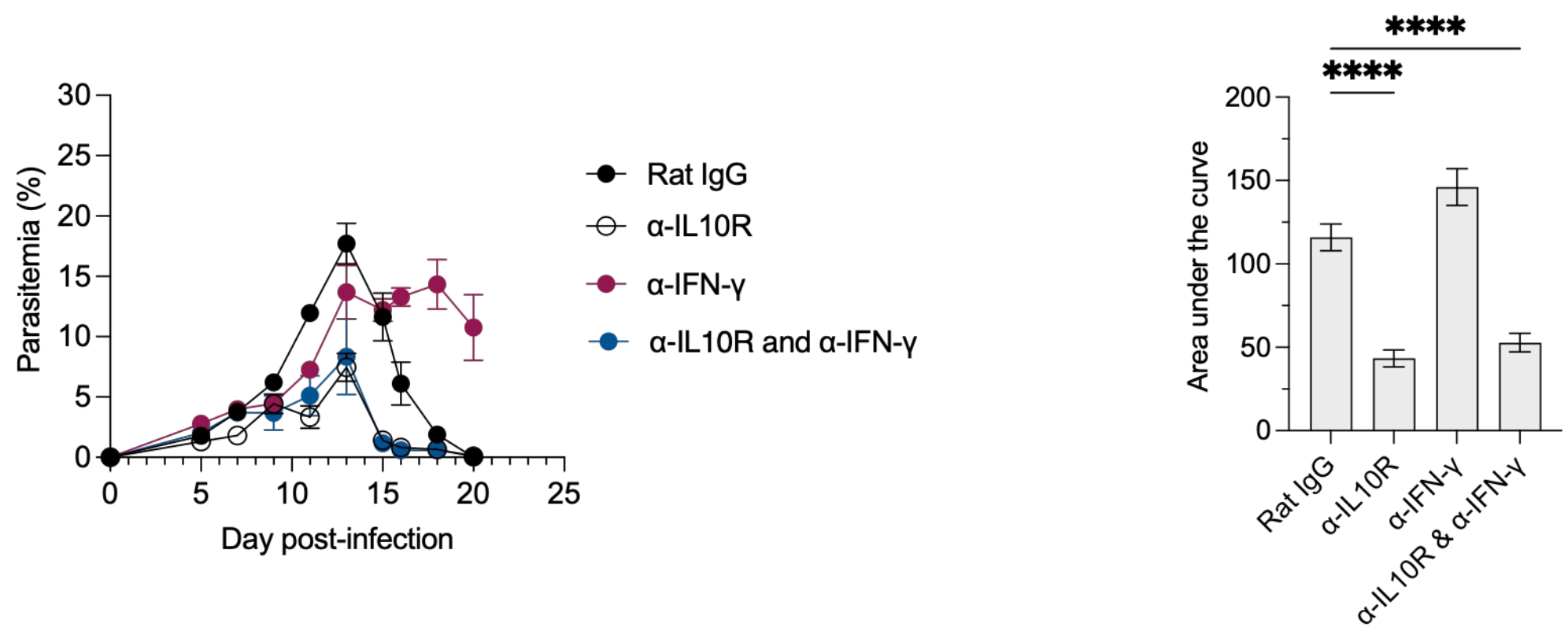

Fig S5. WT mice were infected i.p. with 10^5 *P. yoelii* 17X pRBCs. (**A**) Representative parasitemia curve of the primary infection. WT mice were i.p. injected with either 200 μg of Rat IgG, 500 μg anti-IFN-γ, 200 μg anti-IL10R, or both anti-IFN-γ and anti-IL-10R at day 1 p.i. and every 3 days after. WT mice served as controls. (**B**) Area-under-the-curve analysis included data for the entire curve in A. n = 4 mice per group.

Table SI. List of antibodies used for flow cytometry.

Antibody	Fluorophore	Clone	Catalog/Reference #	Company
GL7	AF488	GL7	144612	BioLegend
CD38	AF700	90	102742	BioLegend
T-bet	APC	4B10	644814	BioLegend
CD19	BV510	6D5	115546	BioLegend
CXCR5	BV5605	L138D7	145513	BioLegend
Ly6C	BV650	HK1.4	128059	BioLegend
B220	BV650	RA3-6B2	103241	BioLegend
CXCR6	BV711	SA051D1	151111	BioLegend
PD-1	eFlour450	RMP1-30	48-9981-82	eBioscience
IgM	eFlour660	11|41	50-5700-82	Invitrogen
Fixable viability dye	eFlour780		65-0865-14	Invitrogen
CD11a	FITC	M17|4	101106	BioLegend
CD8	Pacific Blue	53-6.7	100725	BioLegend
IgD	Pacific Blue	11-26c.2a	405712	BioLegend
CD11b	PerCP-Cy5.5	M1|70	65-0112-U100	TONBObiosciences
CD11c	PerCP-Cy5.5	N418	65-0114-U100	TONBObiosciences
Ter119	PerCP-Cy5.5	TER-119	116228	BioLegend
B220	PerCP-Cy5.5	RA3-6B2	65-0452-U100	TONBObiosciences
CD3e	PerCP-Cy5.5	145-2C11	65-0031-U100	TONBObiosciences
CXCR3	PE	CXCR3 - 173	126506	BioLegend
CD138	PE	281-2	142504	BioLegend
Dihydroethidium (DHE)	PE		104821-25-2	Sigma-Aldrich
Fas	PE-Cy7	Jo2	557653	BD Pharmingen
CXCR6	PE-Cy7	SA051D1	151119	BioLegend
IFN-gamma	PE-Cy7	XMG1.2	60-7311-U100	TONBObiosciences
CD4	redFlour 710	RM4-5	80-0042-U100	TONBObiosciences
CD44	v500	IM7	85-0441-U100	TONBObiosciences
